# Psychological Adaptive Mechanism Maturity Predicts Good Outcomes in Treatment for Refractory PTSD

**DOI:** 10.3389/fpsyg.2021.718451

**Published:** 2021-09-30

**Authors:** Thomas Beresford, Lawrence Wahlberg, Daniel Hipp, Patrick J. Ronan

**Affiliations:** ^1^Rocky Mountain Regional Veteran Affairs Medical Center, VA Eastern Colorado Health Care System, Aurora, CO, United States; ^2^Department of Psychiatry, School of Medicine, University of Colorado, Aurora, CO, United States; ^3^Sioux Falls VA Health Care System, Sioux Falls, SD, United States; ^4^Sanford School of Medicine, University of South Dakota, Vermillion, SD, United States

**Keywords:** adaptive mechanisms, ego defense, PTSD, refractory, residential treatment

## Abstract

**Background:** Post-traumatic Stress Disorder (PTSD) severity follows a bell-shaped curve ranging from mild to severe. Those in the severe range often receive the most intensive treatments, including targeted residential rehabilitation stays. These are expensive and welcome ways to improve their effectiveness. We hypothesized that positive change among subjects treated in a 45-day residential rehabilitation format would be associated with the maturity levels of measurable Psychological Adaptive Mechanisms (PAMs), alternately ego defense mechanisms.

**Methods:** In this association study, adult male patients (*N* = 115) with a history of combat related PTSD treated in a residential rehabilitation setting completed the Defense Style Questionnaire (DSQ) on admission, as well as the Post-Traumatic Stress Disorder Checklist-Military Version (PCL-M) and the Mississippi Scale for Combat-Related Post-traumatic Stress Disorder (M-PTSD) on admission and again at discharge. This allowed prospectively calculated change scores on each of the PTSD measures for each patient. The change scores allowed association testing with averaged admission DSQ scores using Pearson's correlation probability with significance held at *p* < 0.05.

**Results:** As hypothesized, averaged individual Mature scores on the DSQ were associated with improved change scores on both the PCL-M (*p* = 0.03) and the M-PTSD (*p* = 0.04). By contrast neither averaged DSQ Neurotic or Immature scores associated significantly with either PTSD scale change scores.

**Conclusion:** These results, the first of their kind to our knowledge, suggest that patients presenting with predominantly Mature level PAMs are likely to benefit from residential rehabilitation treatment of PTSD. By contrast, those presenting with Neurotic or Immature PAMs predominantly are less likely to encounter positive change in this type of treatment. Although residential treatment is often reserved for the most refractory PTSD cases, it appears that those endorsing Mature level PAMs will make use of residential treatment whereas other forms of treatment may be better suited to those with Neurotic and Immature adjustment mechanisms.

## Introduction

Post-traumatic stress disorder (PTSD) occurs in response to sustained overwhelming stress. Viewed another way, it may present when stressful experiences occur in such strength and duration as to overwhelm the human Psychological Adaptive Mechanisms (PAMs) deployed to manage the high stress level. An immediate reaction to stress is the perception of a threat to one's conscious equilibrium. This in turn leads to a physiological increase in anxiety. Anxiety may be appreciated either in the form of impending disaster or unrelenting doom. Physiological anxiety may be considered as a signal that the human organism is under stress, although the signal may be an unpleasant one. Severe or repeated stresses, or both, may result in chronic anxiety related to the PTSD symptoms including hypervigilance and quick reactivity to incoming threats, real or perceived.

**Figure 1:**
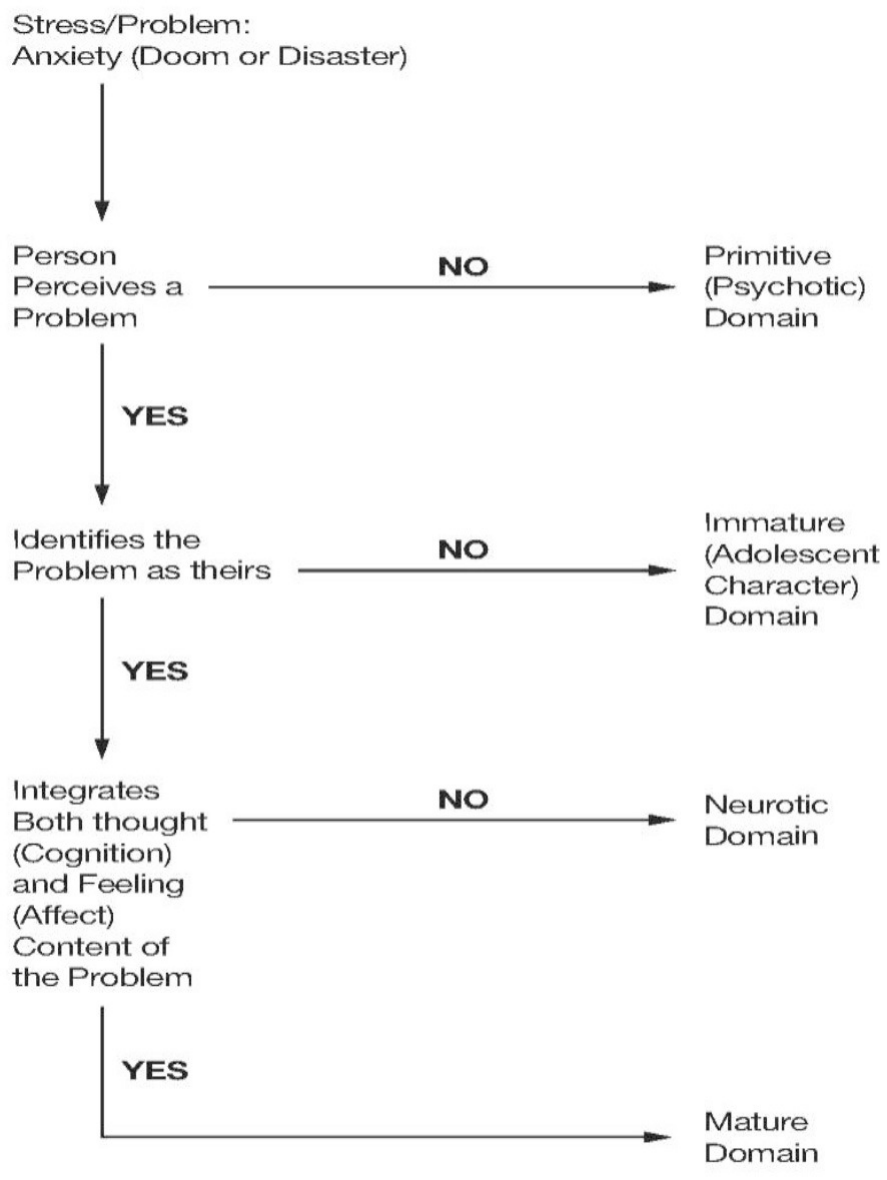
Recognition algorithm for psychological adaptive mechanism domains. Source: Table 3.1 from Beresford (2012, p. 28). Reprinted with permission.

The study of PAMs (Vaillant, 1993; Beresford, 2012) has established that adaptive behaviors occur on a continuum ranging from Primitive, inflexible responses through a hierarchy culminating in Mature, flexible behaviors. Figure 1 presents a clinical algorithm that depicts the differences among Vaillant's four domains, moving from Primitive through Mature. Seen in this manner, a PAM model provides considerable individual variation in observed behaviors occurring in response to stressful situations. The severity of PTSD itself can theoretically be moderated by the relative maturity of adaptive behaviors in response to same or similar stresses. Recent studies have advanced the notion that PTSD exists on a continuum, therefore (Shalev et al., 2017).

With this in mind, we asked whether persons in a residential treatment program for PTSD might have different outcomes based on the relative maturity of their PAMs? We hypothesized that residential treatment improvement would be empirically linked to PAM maturity in a positive manner—the more Mature the subject's adaptive mechanisms, the better residential treatment outcome.

## Methods

### Participants and Procedures

Participants included 115 adult (>18 years) male veterans who completed treatment in the RMRVAMC's Residential PTSD program in 2008. Ongoing PTSD symptoms refractory to standard outpatient treatment and noted to require intensive treatment indicated admission to the residential unit. In all cases, patients admitted for treatment were required to be alcohol and substance free for 30 days prior to admission. This was verified by alcohol and drug screening prior to admission. Data on clinical variables, including PTSD symptom severity, depression, and anxiety were gathered on admission and discharge as part of the program's standard 45-day treatment regimen. Veterans were informed that the data collected would be analyzed to facilitate a quality assurance effort pertaining to the program's treatment effectiveness. The data from clinical intake and discharge testing were entered into a secure electronic database by quality assurance staff. All of the cases were given numerical codes prior to data analysis in order to ensure participants' anonymity and confidentiality. Last, electronic record reviews were conducted to determine veterans' demographic information.

### Study Measures

#### Post-traumatic Stress Disorder

##### The Mississippi Scale for Combat-Related Post-traumatic Stress Disorder

The Mississippi Scale for Combat-Related Post-traumatic Stress Disorder (M-PTSD, Keane et al., 1988) is a 35-item self-report measure of combat-related PTSD symptoms in veterans. Responses are given on a five-point Likert-type scale; low scores indicate little or no evidence of PTSD symptoms and higher scores indicate more severe PTSD symptomatology. Change scores indicate movement in symptom endorsement with movement toward lower scores indicating improvement. Item scores are summed and range from 35 to 175, with scores above 107 suggesting that the respondent is experiencing clinically significant symptoms of combat-related PTSD. The M-PTSD is widely used and has been shown to have excellent internal validity and reliability (Keane et al., 1987).

##### The Post-traumatic Stress Disorder (PTSD) Checklist-Military Version

The Post-Traumatic Stress Disorder Checklist-Military Version (PCL-M, Hoge et al., 2014) is a 17-item self-administered survey designed to give a preliminary assessment of the presence and severity of PTSD symptoms, as defined by the *Diagnostic and Statistical Manual of Mental Disorders* (APA, 2000, 2013). Respondents are asked to rate the extent to which they experienced PTSD-related symptoms over the past month, using a five-point Likert-type scale, ranging from 1 (not at all) to 5 (extremely). Item scores are summed to provide a total PTSD symptom severity score, with higher scores indicating more severe PTSD. The PCL-M is a widely used measure of combat-related PTSD symptoms and has been shown to have very good internal consistency (Ruggiero et al., 2006) and strong convergent validity, as demonstrated by its positive correlation with other measures of PTSD, such as the M-PTSD (*r* = 0.85–0.93), Impact of Event Scale (*r* = 0.90), and Clinician-Administered PTSD Scale (*r* = 0.79) (Yeager et al., 2007; Keen et al., 2008).

#### Psychological Adaptive Mechanisms

##### Defense Style Questionnaire

Each subject filled out printed versions of the 40-item Defense Style Questionnaire (DSQ) (Andrews et al., 1993) on admission. The DSQ was designed for recognition and quantitative measurement of ego-defense mechanisms, alternatively known as Psychological Adaptive Mechanisms or PAMs (Beresford, 2012). These refer to observable behavioral strategies by which individuals adapt to the stresses they encounter in their lives (Vaillant et al., 1986). The DSQ contains 40 itemized statements about what a person does in a difficult situation. The subject is asked to endorse each specific statement on a nine-point Likert-type scale. The 40 items reflect 20 adaptive styles, with two items for each on the scale. Each two respective item scores are averaged, resulting in a mean score for each PAM. Based on Vaillant's empirical work (Vaillant, 1985), the DSQ scores for specific mechanisms are then grouped in an *a priori* fashion into three, rather than Vaillant's four, Domains of psychological adaptive styles: Immature (including Primitive), Neurotic, and Mature. Combined in this way, investigators may then compute category means for each of the three Domains for each study subject.

### Statistical Approach

Tests of association provided the method of assessing the relationship between PAM maturity on admission and improvement each of the two PTSD scales given first at admission and repeated at the end of the 45-day rehabilitation unit stay. For the PTSD scales, computed change scores—admission score minus discharge score—provided a measure of change for each individual. Pearson's *r* coefficient tested the statistical associations between the individual mean admission DSQ scores for each of the three Domains with the change scores recorded for each of the PTSD scale scores, independently. Probability reached significance at the *p* < 0.05 level.

Calculations of mean scores for both PTSD measures and for each of the three PAM Domains allowed group comparisons among each of these measures for purposes of assessing overall PTSD symptom and DSQ Domain frequencies. Student's *t*-test allowed statistical calculation of mean differences with probability judged significant at *p* < 0.05.

## Results

### Demographic Characteristics

Of the 115 veterans for whom data were collected, all were adult (>18 years) male veterans, ranging in age from 27 to 75 (*M* = 54.84, *SD* = 13.12) (Table 1). Ethnic data were available from review of the participants' electronic medical records in 109 of the cases. Ethnic breakdown yielded 58.7% (*N* = 67) of veterans identified as White, 28.4% (*N* = 33) as Hispanic, 9.2% (*N* = 10) as African American, and 2.8% (*N* = 3) as Native American. One veteran identified as multiracial (0.9%) and data were missing for the remaining six participants. Participants' level of education ranged from 9.0 to 20.0 years (*M* = 13.34, *SD* = 1.96).

**Table 1:** Demographic characteristics.

	***N***	**Mean or %**
Gender		
Male	115	100%
Age	111	54.85 ± 13.11
Education	109	13.34 ± 1.96
Race/Ethnicity	109	
Caucasian	64	58.70%
Hispanic	31	28.40%
African American	10	9.20%
Native American	3	2.90%
Biracial/Multiracial	1	0.90%

### PTSD Change Scores by Adaptive Mechanism Domain

For the whole sample, mean calculation yielded a PCLM average score of 64.9 ± 19.3 and 133.6 ± 21.0 average Mississippi score, both signifying PTSD (Table 2). On average, PTSD change scores showed mild improvement—a score decrease of about six points on each scale—between admission and discharge: PCLM at −6.1 ± 13.6, and Mississippi at −6.4 ± 21.3. The standard deviations suggest wide variations of outcome over the course of the treatment program. A *t*-test of the mean differences between the two measures was non-significant. For clinical perspective, US Department of Veterans Affairs' National Center for PTSD recommends “using 5 points as a minimum threshold for determining whether an individual has responded to treatment and 10 points as a minimum threshold for determining whether the improvement is clinically meaningful.” See https://ipgap.indiana.edu/documents/ptsd_intruments/pcl-manual.pdf.

**Table 2:** Mean difference and association probabilities (*n* = 115).

**PAM domains**	**Mature**	**Neurotic**	**Immature**
mean frequency vs. mature domain (*t*-test)		*p* < 0.03	*p* < 0.0001
Mississippi	*p* < 0.03	ns	ns
PCL-M	*p* < 0.04	ns	ns

*Within DSQ domains, and vs. Mississippi and PCL-M*.

At the time of admission, the average DSQ endorsements on the nine-point scale over the three adaptive Domains, respectively, exhibited much smaller standard deviations: Immature 5.2 ± 1.1, Neurotic 5.5 ± 5.2, and Mature 4.4 ± 1.3. Student's *t*-test of the means revealed differences in endorsement patterns across the Domains. The subjects endorsed Mature adaptive mechanisms significantly less often than they did the Immature (*p* < 0.001) or the Neurotic (*p* < 0.03) PAMs.

To define the relationship, if any, between change scores on the PTSD measures and PAM Domain endorsement we used Pearson's correlation coefficient. Change scores on both the PCLM and the Mississippi scales were positively and significantly associated with Mature Domain endorsement, *r* = 0.20, *p* < 0.03 and *r* = 0.19, *p* < 0.04, respectively. By contrast, there was no statistical correlation between the change scores and either Neurotic or Immature Domain endorsement.

## Discussion

### Conclusion

Psychological Adaptive Mechanism assessment appeared to sort those with improved PTSD vs. those with no improvement, on average, in this association study, validating our hypothesis. This finding raises the possibility that systematic PAM assessment may be useful in directing residential treatment resources to those refractory cases who can benefit most from them. Testing this new hypothesis appears best done in a more complex, prospective design of PTSD treatment outcome, expanding on that used in this instance. To do so, however will likely require further inquiry as described below.

### Limitations

This study assessed a relatively small, exploratory sample of cases admitted after failing outpatient treatment for PTSD. No prior treatment descriptors were available from which to analyze other potential factors contributing to outcome or prognosis. Further study of the question of PAM maturity and PTSD outcome will require larger patient samples and more in-depth collection of pertinent variables than can be offered in the scope of the present study.

The present report describes a consecutive sample of the severe PTSD cases in whom outpatient treatment did not offer sufficient symptom resolution. This was done for quality assurance purposes rather than for broader scientific inquiry. Future studies may include a wider selection of post-combat persons with PTSD, for example, whose treatment spans outpatient as well as residential modalities. Assessment on such instruments as the DSQ can allow a basis for random assignment of outpatient vs. residential treatment.

### Uses

While useful statistically in a sample of this nature, the DSQ in its present form does not offer the clinician a way of characterizing individual persons with greater or lesser endorsement of the three domains mentioned. That is, at present there is no “cut point” measure that can offer the clinician a way of sorting the three DSQ Domains in real time such that, with confidence, they could be assigned to one or another treatment group.

Others point out that assessing PAMs, mechanisms that reside in the unconscious until needed, cannot be accomplished directly using self-reports. Vaillant's original investigations addressed this by reporting empirical analyses of action vignettes describing observable behaviors in the face of challenging circumstances. Independent raters then classified each vignette as best exemplifying one of the adaptive mechanisms from Vaillant's original glossary (Vaillant, 1971) of 18 separate defense/adaptive mechanisms. The independent ratings allowed assessments of reliability. Perry and colleagues later used the same method, substituting recorded video interviews for the vignettes (Perry and Cooper, 1989).

About the same time, Andrews and colleagues developed the DSQ as a brief, self-report survey that took less time to administer (Andrews et al., 1993). As in the present report, this approach sacrificed individual case characterization for greater ease of data collection in the aggregate. It is important to note that the DSQ, contrasted to our PAM algorithm used for clinical purposes, construes Vaillant's defenses differently: three Domains, with Primitive and Immature combined in the DSQ as compared to the original four Domains in the algorithm.

Perry and colleagues developed the Defense Mechanism Rating Scale (DMRS) as another approach to reliable assessment (Perry, 1990; Di Giuseppe et al., 2020). Di Giuseppe and associates took this further using a Q-sort procedure, the DMRS-Q (Di Giuseppe et al., 2014). The same item endorsement approach characterizes these assessments and results in a complex reading of a panoply of defenses in individual cases.

Two concerns persist, however. First, these approaches still rely on a series of items that may be time-consuming to collect and require available, specialized software for analysis. The results offer spectra of defensive tendencies rather than definitive statements of specific adaptive strategies. Second, the items themselves depend on statements of what a person would or might do in a particular situation; corroboration must come from external sources such as reports from significant others or from a treatment team's serial, multiple observations. As Vaillant has pointed out, what people do generally offers a far more reliable indicator of adaptation than what they say (Vaillant, 1977).

The principal investigator of the present report adopts another approach for clinical use based on the application of traditional medical assessment and diagnosis (Beresford, 2012, 2014). In this method, a clinician trained in PAM recognition takes a clinical history of behavioral adaptations to current stresses in their lives, focusing on actions that can be observed. From these histories, the clinician uses the brief algorithm in Figure 1 (Beresford, 2014) that sorts PAMs by Domain using Vaillant's original glossary, the simplest available (Vaillant, 1993). From this the knowledgeable clinician can arrive at an accurate formulation of operant PAMs in an individual case. One limitation occurs in the need to train clinicians in this method. Both the method and the algorithm can be taught, however, and potentially offer more efficiently gathered, useful evidence for clinical decision-making.

This study offers the hope for more directed, effective treatment for patients suffering from the more refractory forms of PTSD. We have shown that those who endorse Mature PAMs more frequently responded to the residential treatment whereas those endorsing Immature, or Neurotic PAMs received little benefit from such treatment. Explanations of the positive effects of the Mature PAMs in this setting, vs. the null effect of the other two Domains, deserve further investigation. For now, in reference to the distinctions presented in Figure 1, when faced with a stress, humans are at their most adaptable when they can (1) recognize a problem, (2) note it as one over which they have control, (3) identify the painful thoughts and the painful feelings it brings, and (4) integrate both feelings and thoughts in the interest of a flexible solution. By contrast, ignoring a problem, ascribing it to others, and finding an inability to reconcile the associated painful thoughts and feelings, all limit the options available for its resolution.

In the context of PTSD treatment assignments, further study may also determine whether this correlation between PAM maturity and PTSD improvement can be used as an *a priori* screen to help determine a treatment plan. It may be, for example, that growth toward, or reconstitution of, Mature adaptive mechanisms is facilitated by PTSD treatment, even in refractory cases. These, or other possible approaches, for example an artificial intelligence (AI) application of the Q-sort/DMRS process, will require further development and testing in order to arrive at a workable mechanism for assignment of appropriate patients to the more resource intensive PTSD treatment modalities.

## Data Availability Statement

The raw data supporting the conclusions of this article will be made available by the authors, without undue reservation.

## Author Contributions

All authors listed have made a substantial, direct and intellectual contribution to the work, and approved it for publication.

## Funding

All the authors receive support for their work from the Department of Veterans Affairs; TB and PR receive partial support from Department of Veterans Affairs Merit Review Award, I01BX004712.

## Author Disclaimer

The views expressed in this article are those of the authors and do not necessarily reflect the position or policy of the Department of Veterans Affairs or the United States government.

## Conflict of Interest

The authors declare that the research was conducted in the absence of any commercial or financial relationships that could be construed as a potential conflict of interest.

## Publisher's Note

All claims expressed in this article are solely those of the authors and do not necessarily represent those of their affiliated organizations, or those of the publisher, the editors and the reviewers. Any product that may be evaluated in this article, or claim that may be made by its manufacturer, is not guaranteed or endorsed by the publisher.
